# QTL mapping and key candidate gene identification of resistance to frogeye leaf spot (*Cercospora sojina*) in soybean

**DOI:** 10.3389/fpls.2026.1773160

**Published:** 2026-05-11

**Authors:** Xiaonan Wang, Qichao Zhao, Keyun Wu, Bo Hu, Pengfei Xu, Yihan Huang, Zhimin Dong, Shuzhen Zhang, Wen-Xia Li, Liangliang Yao, Hailong Ning

**Affiliations:** 1 Key Laboratory of Soybean Biology, Ministry of Education, Key Laboratory of Soybean Biology and Breeding/Genetics, Ministry of Agriculture, Northeast Agricultural University, Harbin, China; 2 Northeast Agricultural Research Center of China, Jilin Academy of Agricultural Sciences, Changchun, China; 3 Academy of Agricultural Sciences of Heilongjiang, Jiamusi, China

**Keywords:** candidate genes, frogeye leaf spot, molecular breeding, QTL, soybean

## Abstract

Soybean frogeye leaf spot (FLS) is a global fungal disease that adversely affects both the yield and quality of soybean. The available resistant loci and genes for FLS are limited, thereby constraining the molecular breeding of soybean for FLS resistance. In this study, a recombinant inbred lines (RIL) population (RIL3613), derived from the cross of Dongnong L13 and Heihe 36, was utilized to analyze the genetics of resistance and identify resistant loci. By combining the relative lesion area (RLA) data from each individual of RIL3613 collected across two environments with a high-density bin genetic map, a total of 19 quantitative trait loci (QTL) were identified, distributed across 9 chromosomes, with phenotypic variation explained (PVE) ranging from 1.12% to 18.93%. Notably, 8 QTL were consistently located in both environments, prompting a search for candidate genes within these stable QTL. Through parental sequence variation analysis, expression level determination, haplotype analysis, and gene function annotation, one gene (*Glyma.15G245300*) within qFLSm-15-1, which encodes cytochrome P450, was selected as a candidate gene. This study identified several novel loci and genes that may enhance molecular breeding efforts aimed at improving resistance to FLS in soybean.

## 1 Introduction

Soybean frogeye leaf spot (FLS), caused by *Cercospora sojina* K. Hara, is a prevalent disease that significantly diminishes soybean yields. Regions characterized by high temperatures and humidity are particularly prone to FLS outbreaks. The primary symptoms of this fungal pathogen include small, frogeye-like lesions on the leaves, which impair photosynthesis and reduce chlorophyll content, potentially resulting in premature leaf drop. Additionally, the stem, pods, and seeds may also become infected. In years of severe outbreaks, the disease can lead to a reduction in soybean seed yield exceeding 60% (Akem and Dashiell, 1994). The most cost-effective and efficient strategy for controlling FLS involves leveraging natural genetic resistance to develop new resistant cultivars.

Multiple physiological races of *C. sojina* exhibit variability in both pathogenicity and geographical distribution. In 1981, researchers in the United States identified over 10 physiological races (Phillips and Boerma, 1981), while subsequent studies documented the existence of 22 races (Mian et al., 2008). Races 11 and 12 have been reported in Argentina (Scandiani et al., 2012). Recently, the prevalence of races 7 and 15 has risen in China.

Soybean FLS is predominantly governed by a single dominant resistance gene, referred to as *Rcs* (Sun et al., 2022). The genes *Rcs1* and *Rcs2*, discovered in the American soybean varieties ‘Lincoln’ and ‘Kent’, respectively, provide resistance to physiological races 1, 2, and 5. *Rcs3*, identified in ‘Davis’, offers resistance not only to race 5 but also to all known FLS races in the United States and Brazil (Probst et al., 1965; Yorinori, 1992). Furthermore, a gene designated *Rcs7* imparts resistance to Chinese race 7 (Zou et al., 1999). Ongoing research has led to the identification of several novel loci or genes.

To fine-map the novel resistance genes in PI594774 and PI594891 on chromosome 13, recombinant individuals were identified within an F_2:3_ population derived from the cross between PI594891 and PI594774. The genomic region was refined to 72.6 kb, which encompasses five annotated genes. Kompetitive Allele Specific PCR (KASP) assays were conducted utilizing SNPs from three candidate genes (Pham et al., 2015). Sharma and Lightfoot (2014) performed QTL mapping for the American physiological race 2 of FLS, identifying two major QTLs located near Satt319 on chromosome C2 and Satt632 on chromosome A2. Gu et al. (2021) executed a genome-wide association study employing 30,890 SNPs to analyze resistance to physiological race 15 in Chinese soybean germplasm, identifying four SNPs associated with FLS resistance. Sun et al. (2022) assessed resistance to Chinese FLS race 1 through GWAS and SNP genotyping in 183 soybean accessions, revealing 13 SNPs significantly linked to resistance.Marker-assisted selection (MAS) can enhance the efficiency of traditional breeding methods; however, the development of molecular markers associated with resistance to FLS remains limited. Mian et al. (1999) mapped *Rcs3*, a gene that confers resistance to physiological race 5 of soybean FLS in the United States, and identified a close linkage to the SSR marker Satt244, followed by Satt547, which is situated 1.5 cM from *Rcs3*. Yang et al. (2001) localized the resistance gene *RcsPeking* in the cultivar ‘Peking’ using AFLP and SSR markers, demonstrating that it is positioned between the markers AACCTA178 and Satt547. Their findings indicated that *RcsPeking* is confined to a 2.1 cM interval between AACCTA178 and Satt244, with distances of 1.1 cM and 1.0 cM from Satt244 and AACCTA178, respectively.

Breeding novel cultivars resistant to FLS is of paramount importance; however, the loci, genes, and molecular markers available for molecular breeding are currently limited. Consequently, the primary objective of this study was to identify new resistance loci and genes. Phenotypic data were collected across two environments, and QTLs associated with resistance to FLS were identified using a bin-based genetic map. The resistance-related QTLs and candidate genes identified in this study can be employed in molecular breeding, thereby enhancing disease resistance in soybean.

## 2 Materials and methods

### 2.1 Plant materials and field design

An experiment was conducted using the recombinant inbred line population RIL3613, derived from a cross between Dongnong L13 (FLS-sensitive) and Heihe 36 (FLS-resistant), known for their differing FLS resistance levels. The RIL3613 population, comprising 120 individuals, was generated through the single seed descent method in Harbin, Heilongjiang Province (E 126.63°, N 45.75°). In 2021, field trials with this population were carried out in Xiangyang (XY, E 126.63°, N 45.75°) and Acheng (AC, E 127.63°, N 45.82°), at the experimental stations of Northeast Agricultural University. A randomized complete block design with three replications per site was employed, featuring a plot length of 3 m, row spacing of 0.7 m, and plant spacing of 0.07 m. Field management practices adhered to standard soybean production protocols.

### 2.2 Phenotypic assessment of leaf lesion area

The mixed races of three predominant FLS physiological species in Heilongjiang Province, namely race 1, race 7, and race 15 with the ratio of 1:1:1, were employed to assess the resistance of genetic materials with. The original bacteria were sourced from the Jilin Academy of Agricultural Sciences. The bacteria were cultured on PDA medium, activated, and cultivated in an incubator at 27 °C for 7–15 days. Subsequently, secondary propagation was conducted using sterilized sorghum medium, which was then cultivated at 27 °C for 15 days. The mycelium present on the sorghum surface was moistened with distilled water, manually agitated to create a spore suspension, filtered through a strainer, and diluted to a concentration of 1 × 10^7^ spores/mL by the addition of water.

Due to the local climatic conditions and the growth stage of soybeans, inoculation was carried out during the R3-R4 growth stage when the soybean leaves were fully expanded and most vulnerable to “frogeye leaf spot” infection. Two inoculations were carried out in Xiangyang on August 4 and August 14, 2021, and in Acheng on August 11 and August 21, 2021. Disease incidence was evaluated 20 days post-inoculation when soybean leaves were fully developed. Five plants showing uniform disease symptoms were chosen from each plot. Subsequently, one leaf per plant was collected from the top, middle, and bottom canopy positions, photographed, and the lesion count was documented.


$$\text{PDLA}=\frac{\text{Diseased spot area pixel }}{\text{Full leaf pixel}}$$

PDLA: percentage diseased leaf area.

The evaluation of each line was repeated three times, and the mean PDLA value for each line was used in genetic analysis (McDonald et al., 2023).

### 2.3 QTL mapping

The entire RIL3613 population underwent genotyping using the SoySNP660K BeadChip (Beijing Boao Biotechnology, Beijing, China). After applying quality filtering criteria with a minor allele frequency (MAF) > 0.05 and a maximum missing data rate per SNP < 10%, a total of 108,342 SNPs were identified across the 20 chromosomes in this cohort (Belamkar et al., 2016). These SNP markers were utilized for constructing the genetic map. The high-density SNP bin map of the RIL3613 population covers all 20 chromosomes, containing a total of 2225 bin markers. The total length of the map is 2969.84 cM. The number of SNP bin markers on each chromosome ranges from 54 to 177, and the length of each chromosome is between 60.94 and 273.38 cM. The bin-based genetic map (SNPbin map) was previously established by Wang et al. (2022).

Utilizing the genetic map, inclusive composite interval mapping (ICIM-ADD) was applied to identify additive QTLs linked to resistance against PRLA of FLS (Li et al., 2008). QTL mapping was performed across two environments, as well as their mean values. The scanning interval was established at 1.00 cM, the LOD threshold was set to 2.50, and the retention probability for controlled molecular markers was fixed at 0.001.

The phenotypic trait of soybean FLS derived by mixed races was named as FLSm. The QTL were named followed (McCouch, 1997), for example, qFLSm-2–1 represents the first QTL located on chromosome 2.

### 2.4 Prediction of candidate genes

Based on the results of QTL mapping, QTLs consistently identified in both environments were selected for candidate gene prediction. Genes located within the QTL intervals were identified using Phytozome (https://phytozome.jgi.doe.gov). Functional annotation and protein structure information for these genes were obtained from the NCBI database(https://www.ncbi.nlm.nih.gov), while pathway analysis was conducted through the Kyoto Encyclopedia of Genes and Genomes (KEGG; http://www.kegg.jp) and Gene Ontology (GO; http://kobas.cbi.pku.edu.cn/kobas3) enrichment analysis. Collectively, these analyses facilitated the identification of candidate genes associated with resistance to frogeye leaf spot. Potential candidate genes likely involved in the regulation of disease resistance were further prioritized based on sequence variations between the two parental lines, gene functional annotations, and supporting literature evidence.

### 2.5 Haplotype analysis of candidate genes

The DNA of Dongnong L13, Heihe 36, and RIL3613 was individually extracted using the FastPure Plant DNA Isolation Mini Kit (Vazyme Biotech Co., Ltd). Primers for genetic analysis were designed based on the SNP loci identified between the parental strains (**Supplementary Table 1**). The variable sites within the candidate genes were amplified and sequenced in RIL3613 and a germplasm population (GP) comprising 151 varieties(**Supplementary Table 2**). Haplotype analysis was carried out utilizing both genotypic and phenotypic data. The haplotype analysis of the candidate genes was integrated with the phenotypic information on the relative lesion area of the test varieties post-infection with a mixed population of the gray spot disease pathogen. Statistical analysis of significant differences in haplotype associations was performed using GraphPad Prism 8.0.2 software.

### 2.6 Analysis of haplotype distribution

A thorough statistical analysis was conducted to examine the distribution of various haplotypes of the target gene among cultivated, landrace, and wild soybean varieties. This analysis utilized whole-genome resequencing data from a substantial dataset of 2,883 germplasm accessions sourced from the SoyOmics database at the National Bioinformatics Center (https://ngdc.cncb.ac.cn/soyomics/index). The dataset consisted of 1,733 cultivated varieties, 1,048 landraces, and 102 wild soybean accessions. The resultant haplotype frequency distributions were represented in a pie chart created using Excel 2016, enabling straightforward comparative analysis across the three germplasm categories.

### 2.7 Expression mode analysis of candidate genes

To validate the candidate genes, their relative expression levels were assessed through RT-qPCR analysis of leaf samples collected at six time points (0 h, 1 h, 3 h, 6 h, 12 h, and 24 h) post spore suspension application. Total RNA was isolated using the FastPure^®^ Universal Plant Total RNA Isolation Kit (Vazyme Biotech Co., Ltd.), reverse-transcribed into cDNA with the HiScript^®^III All-in-one RT SuperMix Perfect for qPCR (Vazyme Biotech Co., Ltd.). The 20-μL RT-qPCR reaction mixture included 2 μL of cDNA template, 10 μmol/L each of forward and reverse primers, 10 μL of 2×ChamQ Universal SYBR qPCR Master Mix, and 6.4 μL of ddH_2_O. The amplification program comprised an initial denaturation at 95 °C for 30 s, followed by 40 cycles of 95 °C for 10 s and 60 °C for 30 s, with a final dissociation step to verify amplification specificity. *TUA5* served as the reference gene for transcript normalization, and gene expression data were analyzed using the 2^^−ΔΔCt^ method. The primer sequences can be found in **Supplementary Table 3**.

### 2.8 Analysis of promoter elements and protein tertiary structure of candidate genes

The 2,500 bp promoter sequence located upstream of the gene start codon was obtained from the Phytozome database (https://phytozome-next.jgi.doe.gov/). Potential cis-regulatory elements within the promoter region were then predicted using the PlantCARE web server (https://bioinformatics.psb.ugent.be/webtools/plantcare/html/). The results of these predictions were visualized with TBtools-II (Toolbox for Biologists, version 2.119).

The amino acid sequences derived from the gene were obtained from the Phytozome database(https://phytozome-next.jgi.doe.gov/) to access annotated and curated genomic information. Protein tertiary structures for various genotypes of the gene were predicted utilizing the AlphaFold3 server(https://alphafoldserver.com/), which employs advanced deep learning techniques for precise structural prediction. Subsequently, the generated structural models were visualized and compared using PyMOL 3.1.6.1, facilitating a thorough examination of conformational variances linked to genetic distinctions.

## 3 Results

### 3.1 Phenotype variations of the resistance to FLS in RIL3613

**Figure 1:**
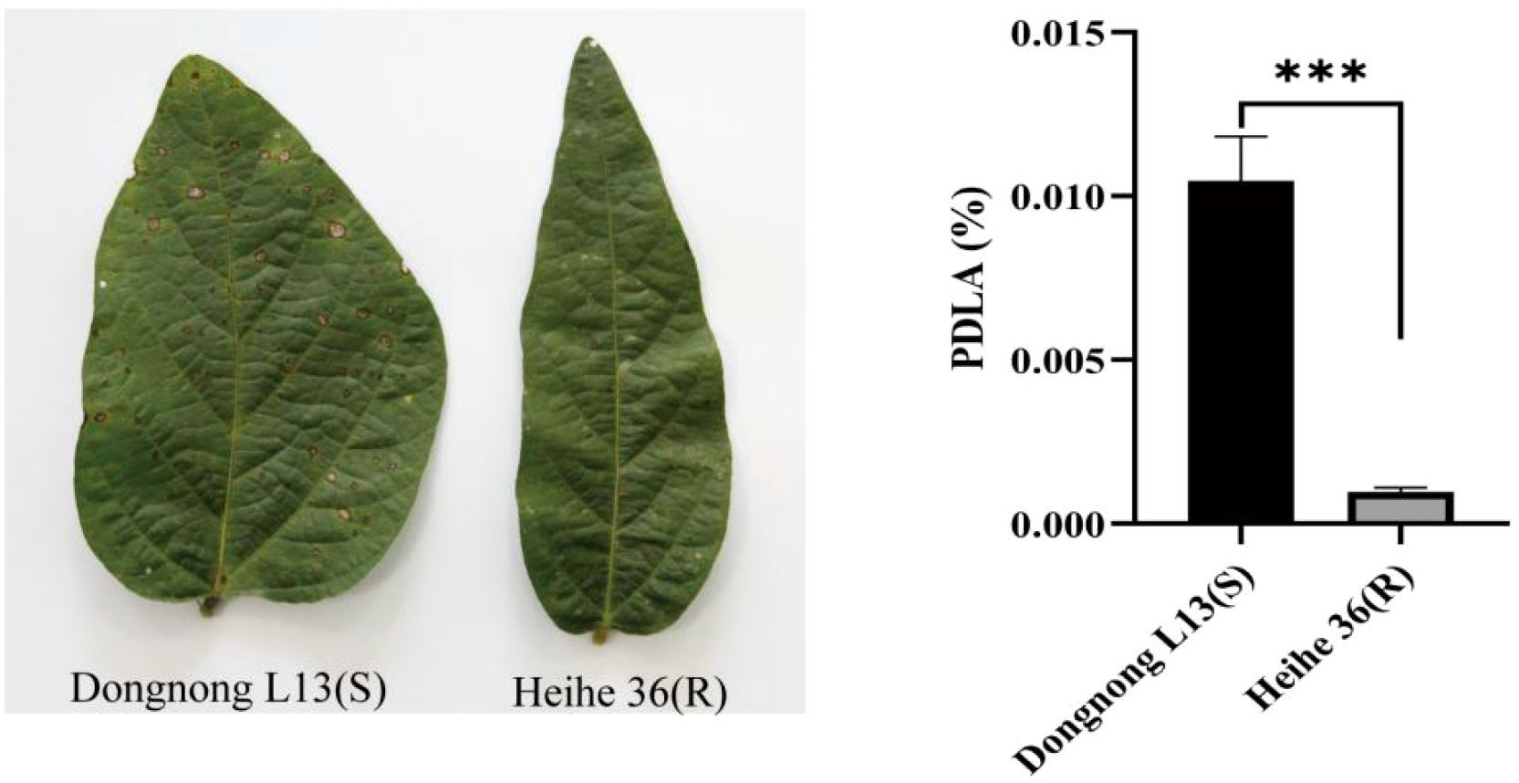
The phenotypic comparison chart and the PDLA bar chart between the resistant parent Hehei 36 (R) and the susceptible parent Dongnong L13 (S). ***P < 0.001 represents the relative expression level.

The phenotypic comparison diagram of the RIL3613 population, which was derived from the susceptible parent Dongnong L13 and the resistant parent Heihe 36, after being sprayed with a mixture of gray leaf spot pathogen, as well as the significance analysis of PDLA, are shown in **Figure 1**. Descriptive statistics, analysis of variance (ANOVA), and heritability estimation were performed on RLA phenotypic data from RIL3613 in two environments using SAS software (**Tables 1**, **2**, **Supplementary Figure 1**). The descriptive analysis revealed a wider phenotypic range in RIL3613 compared to the parental lines, suggesting transgressive segregation for FLS resistance within the population. ANOVA results indicated significant differences for genotype and genotype × environment interaction effects (*p* < 0.01), underscoring substantial genetic variation in FLS resistance and the impact of environmental conditions on resistance trait expression. The broad-sense heritability estimate across both environments was 46.59%, this is consistent with the study by Barro et al. (2023), which indicated that the generalized heritability of FLS resistance was 40%–60%, which is a moderate genetic level. McDonald et al. (2023) precisely located Rcs3 to the 1.15 Mb interval on chromosome 16 of soybean, corresponding to a genetic distance of 2.7 cM; the major marker GSM883 can explain 54%–57% of the phenotypic variation, and is a typical major resistance QTL/gene. This indicates that both genetic factors and environmental factors have an impact on resistance, thereby confirming the necessity of conducting multi-environment tests. signifying that genetic factors contribute to approximately half of the observed phenotypic variation in FLS resistance in soybean under varying environmental circumstances.

**Table 1:** Descriptive statistics for PDLA of frogeye leaf spot resistance in RIL3613 population under two environments.

Env.	Parents^a^			RIL3613					
	Dongnong L13	Heihe 36	Min^a^	Max^b^	Range^c^	Mean^d^	SD^e^	Skew	Kurt
XY	17.66%	3.06%	0.00%	29.52%	29.52%	4.02%	0.039	3.407	1.876
AC	12.45%	2.85%	0.00%	28.80%	28.80%	4.56%	0.047	2.153	2.160

Min^a^, minimum observation of phenotypic data.

Max^b^, maximum observed values for phenotypic data.

Range^c^, Difference between the maximum and minimum values of phenotypic data.

Mean^d^, average of phenotypic data.

SD^e^, standard deviation of phenotypic data.

Skew, Skewness of phenotypic data.

Kurt, The kurtosis of phenotypic data.

**Table 2:** ANOVA and heritability estimation of PDLA in RIL3613 in two environments.

Source	DF	SS	MS	F	Pr>F	*σ* ^2^
Environment	1	0.0101	0.010	4.91	0.0272	
Env(Replication)	4	0.0185	0.005	2.26	0.0619	
Genotype	119	0.7710	0.006	3.16	<.0001	0.0005
Environment*Genotype	117	0.4097	0.004	1.71	<.0001	0.0005
Error	472	0.9678	0.002			0.0021
Total	713	2.1773				
*h* ^2^						0.4659

DF, degrees of freedom.

SS, sum of squares.

MS, mean square.

σ², variance component.

P, significance probability (Pr > F changed to P).

h^2^, generalized heritability of frogeye leaf spot disease relative to spot area in two environments.

### 3.2 QTL underlying resistance to FLS

**Figure 2:**
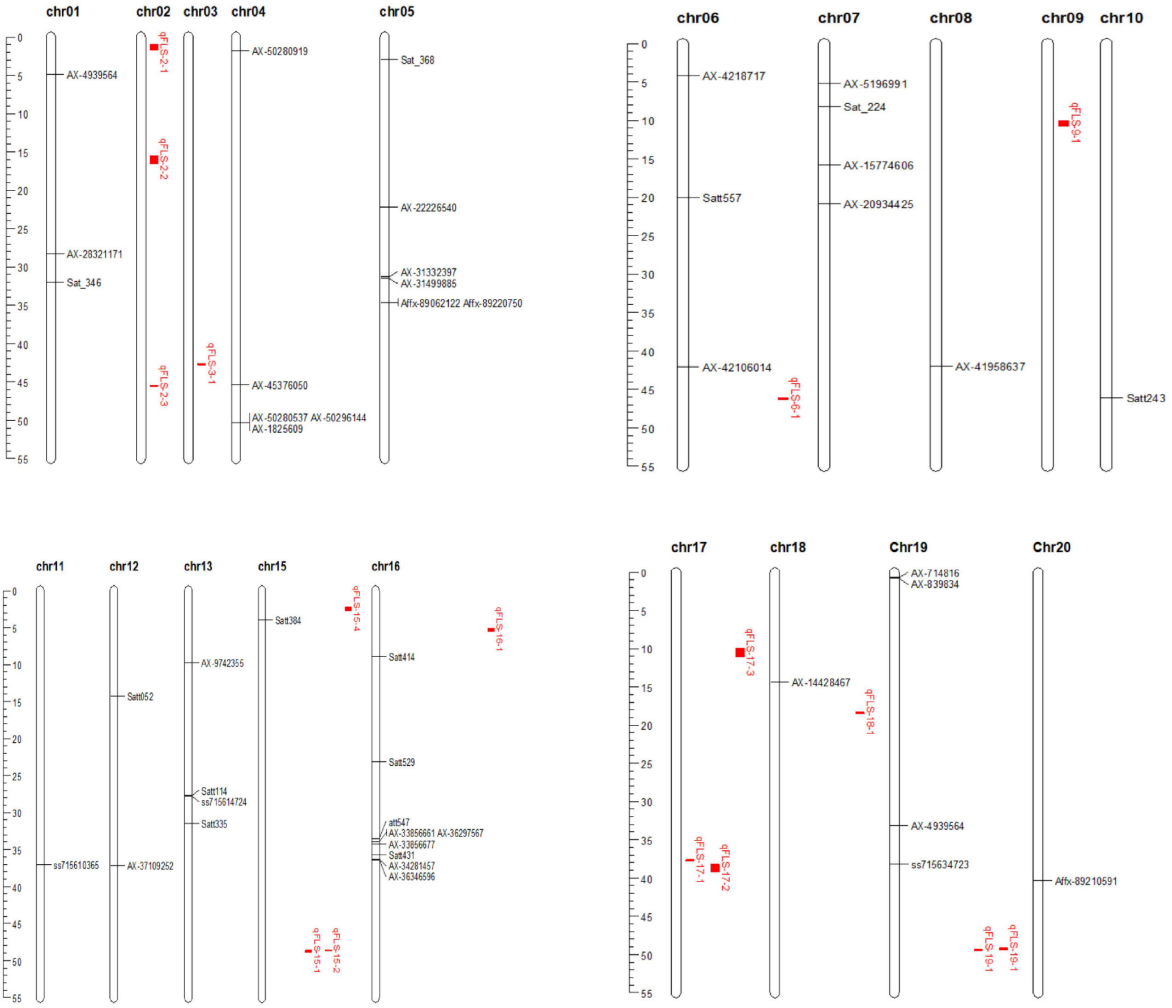
Distribution of QTLs associated with FLS of the present and previous researches.

Nineteen quantitative trait loci (QTL) associated with resistance to FLS were identified in the RIL3613 population and mapped across nine chromosomes (Chr02, Chr03, Chr06, Chr09, Chr15, Chr16, Chr17, Chr18, and Chr19) of the soybean genome. The number of QTL per chromosome varied from 1 to 9, with logarithm of odds (LOD) scores ranging from 2.52 to 23.75 and phenotypic variation explained (PVE) spanning from 1.12% to 18.93%, among them, the phenotypic variation explanation value (PVE) of qFLSm-17–2 was the smallest at 1.12%, while the PVE of qFLSm-18–1 was the largest at 18.93%. Among these, eight QTL—qFLSm-2-1, qFLSm-6-1, qFLSm-15-1, qFLSm-15-2, qFLSm-15-4, qFLSm-16-1, qFLSm-17-2, and qFLSm-18-1—were consistently detected across the AC environments and the mean of the two environments. This consistency indicates their stability and potential reliability as genetic markers for FLS resistance (**Table 3**). Comparative analysis with previously reported loci revealed that qFLSm-15–4 co-localized with regions associated with Chinese Race 7 (Dong et al., 2011; Jiang et al., 2020), while qFLSm-17–2 overlapped with a locus linked to Chinese Race 1 (Sun et al., 2022). The remaining ten QTL represent novel genomic regions associated with FLS resistance identified in this study (**Figure 2**).

**Table 3:** QTL associated with resistance to mixed races of frogeye leaf spot disease.

QTL	Chromosome	Left marker	Right marker	5'-positon	3'-positon	Environment	LOD	PVE (%)	Add	Reported QTL
qFLSm-2-1	2	36c02006	36c02007	1005213	1737807	MEAN	11.11	1.633	0.057	
						AC	13.466	2.68	-0.063	
qFLSm-2-2	2	36c02091	36c02090	15514794	16523887	AC	7.995	3.436	-0.063	
qFLSm-2-3	2	36c02124	36c02123	45454175	45617954	MEAN	3.28	1.482	-0.058	
qFLSm-3-1	3	36c03077	36c03076	42578675	42755661	MEAN	6.17	1.624	-0.051	
qFLSm-6-1	6	36c06108	36c06107	46053890	46327836	MEAN	9.919	1.625	-0.057	
						AC	2.617	2.433	-1.245	
qFLSm-9-1	9	36c09057	36c09058	9947171	10828881	AC	2.52	1.609	-0.031	
qFLSm-15-1	15	36c15158	36c15157	48636935	48862458	AC	12.341	2.888	-0.055	
						MEAN	10.635	1.66	-0.056	
qFLSm-15-2	15	36c15157	36c15156	48573120	48692029	MEAN	10.705	1.66	-0.056	
						AC	12.476	2.917	-0.053	
qFLSm-15-3	15	36c15018	36c15019	5225054	5703286	MEAN	6.683	1.635	-0.056	
qFLSm-15-4	15	36c15005	36c15006	2216621	2645649	MEAN	9.01	1.627	-0.056	Dong et al, 2011; Jiang et al., 2020
						AC	10.642	2.846	-0.058	
qFLSm-15-5	15	36c15006	36c15007	2413555	2739374	MEAN	7.251	1.639	-0.055	
qFLSm-15-6	15	36c15009	36c15010	2795948	3600842	MEAN	6.8	1.628	-0.056	
qFLSm-16-1	16	36c16016	36c16015	5115530	5460013	MEAN	7.968	1.606	-0.051	
						AC	10.683	2.573	-0.043	
qFLSm-17-1	17	36c17077	36c17076	37559338	37816385	MEAN	3.572	1.124	-0.035	
qFLSm-17-2	17	36c17083	36c17082	38236561	39149223	MEAN	10.45	1.641	-0.057	Sun et al, 2022
						AC	12.404	2.686	-0.063	
qFLSm-17-3	17	36c17019	36c17020	9876026	11105395	MEAN	6.386	1.637	-0.056	
qFLSm-18-1	18	36c18041	36c18040	18297976	18525349	MEAN	7.669	1.642	-0.057	
						AC	23.755	18.926	-0.111	
qFLSm-19-1	19	36c19109	36c19108	49343392	49503893	MEAN	5.521	1.637	-0.056	
qFLSm-19-2	19	36c19108	36c19107	49076176	49428145	MEAN	5.783	1.642	-0.056	

LOD, common logarithm of the likelihood rate.

PVE, phenotypic contribution rate of QTL.

### 3.3 Candidate gene for FLS

In the cases of AC, XY, and the average values of the two environments (MEAN), the QTLs that were repeatedly located are defined as stable loci. This study identified eight QTL consistently observed across two environments: qFLSm-2-1, qFLSm-6-1, qFLSm-15-1, qFLSm-15-2, qFLSm-15-4, qFLSm-16-1, qFLSm-17-2, and qFLSm-18-1. These QTL were selected for candidate gene mining due to their stable phenotypic effects and potential genetic significance related to FLS resistance.

A total of 265 genes were identified in the genomic regions of these QTL through the Phytozome database. Subsequently, these genes underwent KEGG and GO functional enrichment analyses. The KEGG annotation revealed that 127 genes were linked to 88 metabolic pathways, prominently in carbohydrate metabolism, amino acid metabolism, biosynthesis of secondary metabolites, lipid metabolism, and signal transduction(**Supplementary Figure 2**), among them, carbohydrate metabolism can provide sufficient energy and carbon skeletons for the biosynthesis of pathogen-related proteins, plant antitoxins, and other defense compounds. The biosynthesis process of secondary metabolites can catalyze the synthesis of substances such as isoflavones and phenols, interfering with the energy metabolism and cell wall synthesis processes of pathogenic bacteria. The related genes may play a role in the plant’s disease resistance process, indicating their probable involvement in defense-related physiological processes. The GO enrichment analysis demonstrated that 153 genes were notably enriched in the three primary ontologies: Biological Process (BP), Cellular Component (CC), and Molecular Function (MF). The most significant -LOG10(P-value) was observed for “proteolysis involved in protein catabolic process” in BP, “lysosome” in CC, and “cysteine-type endopeptidase activity” in MF, respectively, indicating the highest statistical significance in these functional categories(**Supplementary Figure 3**).

A total of 265 genes were identified in 8 stable QTL regions. Through KEGG and GO analysis, we selected all the genes analyzed in the pathways. Based on the gene function annotation information, we found that *Glyma.15G245300* and *Glyma.16G053400* might be involved in the response to plant-pathogen infection. The sequencing results of the parents were imported into the IGV-2.15.2 software to analyze the variations of these two candidate genes on the CDS. It was found that only *Glyma.15G245300* had non-synonymous mutations in both parents, so we chose *Glyma.15G245300* as the key candidate gene for this study for exploration.

### 3.4 Genetic effect of haplotype in *Glyma.15G245300* and molecular marker

**Figure 3:**
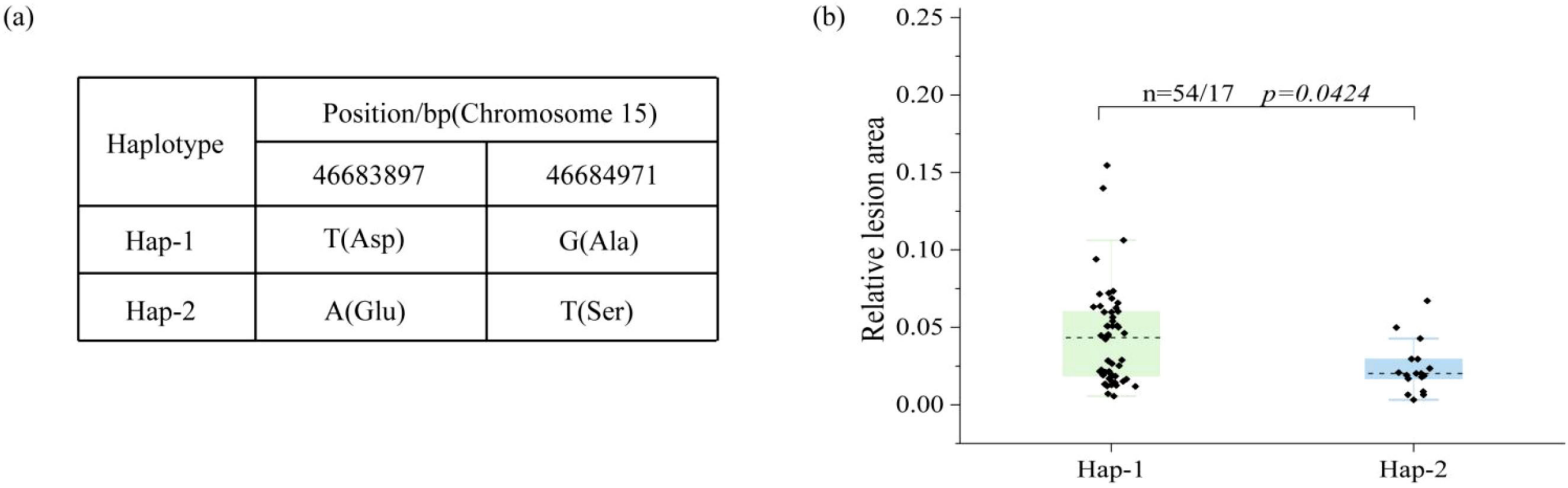
Haplotype analysis of *Glyma.15G245300* in the RIL3613 population. **(a)** The base types of the two haplotypes of the candidate gene. **(b)** The significance analysis of different haplotypes of the candidate gene.

Two non-synonymous variations were found in the CDS region of this gene: a serine to aspartic acid substitution at position 48,677,205 bp and a glutamine to lysine substitution at position 48,676,123 bp. To confirm the genetic impact of the candidate gene *Glyma.15G245300*, resequencing data from the parental lines in the RIL3613 population were analyzed to assess sequence variability in both the promoter and coding regions. While two SNPs were identified in the promoter region, the limited number of mutant lines in the population precluded haplotype-based association analysis. Conversely, two non-synonymous mutations were observed in the coding region, delineating two distinct haplotypes (Hap-1 and Hap-2) within the RIL3613 population (**Figure 3a**). To evaluate potential differences in PDLA among these haplotypes, we developed specific primers targeting the two mutation sites (**Supplementary Table 1**) for amplifying genomic DNA from 71 RIL3613 lines, which were subsequently subjected to Sanger sequencing. The combined analysis of genotypic and phenotypic information demonstrated that PDLA levels were markedly elevated in Hap-1 compared to Hap-2 (**Figure 3b**). These findings suggest a substantial functional disparity between the two haplotypes, with Hap-2 being identified as a beneficial haplotype linked to heightened disease resistance.

**Figure 4:**
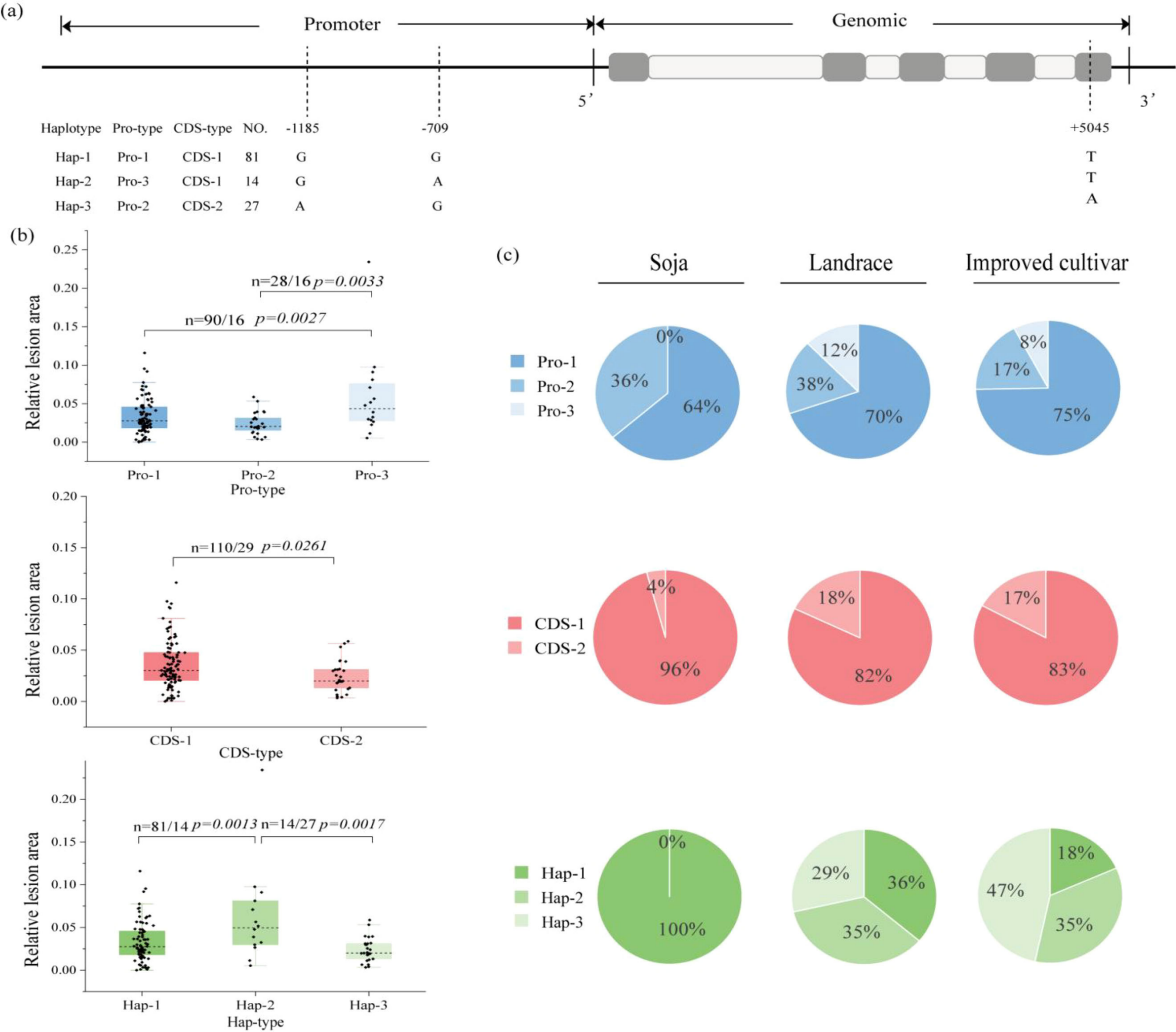
Haplotype analysis of *Glyma.15G245300* in the GP population. **(a)** The variation positions of *Glyma.15G245300* in the promoter and coding region. **(b)** The haplotype analysis of promotor type, CDS type and Haplotype of *Glyma.15G245300* in the GP population. **(c)** The distribution of Promotor type, CDS type and Haplotype of *Glyma.15G245300* in the three major varieties in the GPP population.

To further authenticate and characterize the superior allelic variants of the candidate gene, we analyzed variation sites and haplotypes in a germplasm panel GP consisting of 151 soybean accessions. Within the 2,500 bp promoter region of *Glyma.15G245300*, we identified two SNP variations, allowing for the classification of the promoter into three distinct haplotypes (Pro-1, Pro-2, and Pro-3). Phenotypic assessment revealed significant differences in PDLA among the three promoter haplotypes (*p =* 0.0033/0.0027 < 0.001), with Pro-3 accessions showing notably higher mean PDLA values compared to those carrying Pro-1 or Pro-2. In addition, haplotype analysis was conducted on the sequence of the coding region (CDS). The results showed that the single nucleotide substitution within the 5045th base pair of this gene played a significant role. Specifically, the transition from T to A occurred, resulting in the amino acid changing from aspartic acid (Asp) to glutamic acid (Glu), which is consistent with a non-synonymous mutation found in the RIL3613 population. This coding variation divided into two haplotype categories - CDS-1 and CDS-2. The CDS-1 category contained 110 samples, while the CDS-2 category contained 29 samples. In terms of PDLA, there was a significant difference between these two CDS types (*p* = 0.0261 < 0.05), with the PDLA of CDS-1 samples being higher than that of CDS-2 samples (**Figure 4b**).

In brief, *Glyma.15G245300* contains two SNP variations in the promoter region and one non-synonymous SNP in the CDS region. These variations led to the identification of three promoter haplotypes (Pro-1, Pro-2, Pro-3) and two coding haplotypes (CDS-1, CDS-2). By combining variations from both regions, three composite haplotypes were established: Hap-1 (Pro-1 + CDS-1), Hap-2 (Pro-3 + CDS-1), and Hap-3 (Pro-2 + CDS-2). There was a significant difference in the PDLA values among the three composite haplotypes (*p* = 0.0013/0.0017 < 0.001). The average PDLA of Hap-2 material was significantly higher than that of Hap-1 and Hap-3, which were the susceptible haplotypes. Hap-3 had the lowest average PDLA, being the resistant haplotype. (**Figures 4a, b**). Remarkably, materials carrying the Hap-3 haplotype displayed the lowest PDLA post-FLS infection, indicating reduced disease lesion expansion and suggesting that Hap-3 represents a potentially superior allele linked to enhanced resistance against FLS.

This study, following the classification method of Liu et al. (1991), divided the phenotypes of the GP population into two major categories: susceptible and resistant. 0-3% were classified as resistant, and those above 3% as susceptible. Through haplotype analysis, three composite haplotypes, Hap-1, Hap-2, and Hap-3, were identified. Among them, Hap-2 was the susceptible haplotype, and Hap-3 was the resistant haplotype. We counted the number of resistant/susceptible materials in each haplotype and conducted selection efficiency analysis, which can better apply this molecular marker in breeding practices. The results are shown in **Table 4**. Among the 27 materials carrying Hap-3 (CDS-2 + Pro-2), 19 showed a PDLA of less than 3%, with a selection efficiency of up to 70.4%. This indicates that over 70% of Hap-3 individuals showed resistance, making it the most reliable molecular marker for distinguishing the resistant phenotype. The reverse selection value of the susceptible haplotype Hap-2 is prominent: among the 14 materials carrying Hap-2 (CDS-1 + Pro-3), only 4 showed resistance, with a selection efficiency of only 28.6%. This suggests that this haplotype can be used as a molecular label for the resistant genotype and for reverse elimination of susceptible materials. The results also indicate that compared to single CDS or promoter variations, the selection efficiency differences of composite haplotypes (especially Hap-2 and Hap-3) are more significant, providing stronger discrimination ability for the resistant/susceptible phenotypes, and are more suitable as selection targets for MAS.

**Table 4:** QTL associated with resistance to mixed races of frogeye leaf spot disease.

Haplotype	Hap-1			Hap-2			Hap-3		
	CDS-1	Pro-1	Hap-1	CDS-1	Pro-3	Hap-2	CDS-2	Pro-2	Hap-3
Number	110	90	81	110	16	14	29	28	27
PDLA<3%	54	51	45	54	6	4	20	19	19
Selectivity of efficiency (%)	49.1%	56.7%	55.6%	49.1%	37.5%	28.6%	68.9%	67.8%	70.4%

### 3.5 The distribution of different candidate gene haplotypes in different types of resources

Using resequencing data from 2883 materials in the GPP population, we analyzed the proportions of various haplotypes in wild, local, and improved soybean varieties. The results indicated that the proportion of the Pro-1 haplotype of the *Glyma.15G245300* gene increased progressively across these categories, with values of 64%, 70%, and 75%, respectively. In contrast, the Pro-3 haplotype, which exhibited the highest PDLA, was present in very low proportions across all three categories, specifically 0%, 8%, and 12%. Additionally, the proportion of the CDS-1 haplotype of the *Glyma.15G245300* gene also increased among the three major categories, suggesting that this haplotype has been widely utilized during the processes of variety improvement and domestication. Among the three combined haplotypes, the proportion of Hap-3 increased progressively in wild, local, and improved varieties, while Hap-1 exhibited the highest proportion in improved varieties, accounting for 47% (**Figure 4c**). Analyzing the distribution ratios of the *Glyma.15G245300* gene haplotypes across the three varieties leads to the conclusion that the superior combination haplotype, Hap-1, has been consistently selected and utilized throughout the breeding process to enhance soybean resistance to FLS. Therefore, it can be inferred that *Glyma.15G245300* is a key candidate gene for improving soybean resistance to FLS.

### 3.6 Expression mode of candidate gene *Glyma.15G245300*

**Figure 5:**
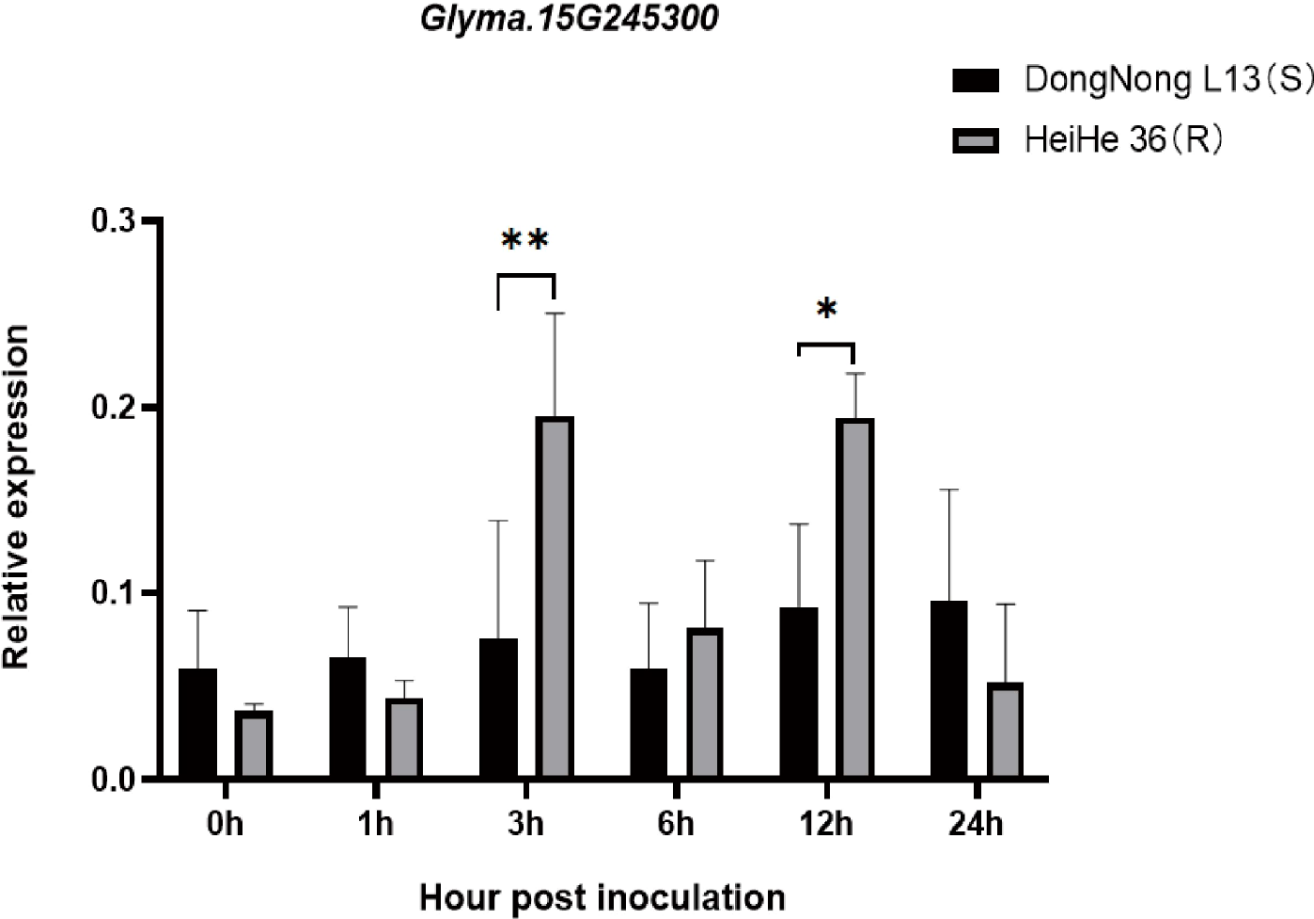
Relative expression of *Glyma.15G245300* gene after the inoculation of FLS. *P < 0.05, **P < 0.01 represents the relative expression level.

Variations in the promoter region led to an examination of the relative expression levels of the *Glyma.15G245300* gene. Parental plants were exposed to a mixed race, and leaf samples were gathered at various time points for RT-qPCR analysis. The induction of *Glyma.15G245300* expression was detected at 3 hours and 12 hours after inoculation, showing a greater relative expression in Heihe 36 than in Dongnong L13 (**Figure 5**). This gene is a pivotal candidate for bolstering soybean resistance to FLS.

### 3.7 Promoter elements and protein tertiary structure of candidate genes *Glyma.15G245300*

**Figure 6:**
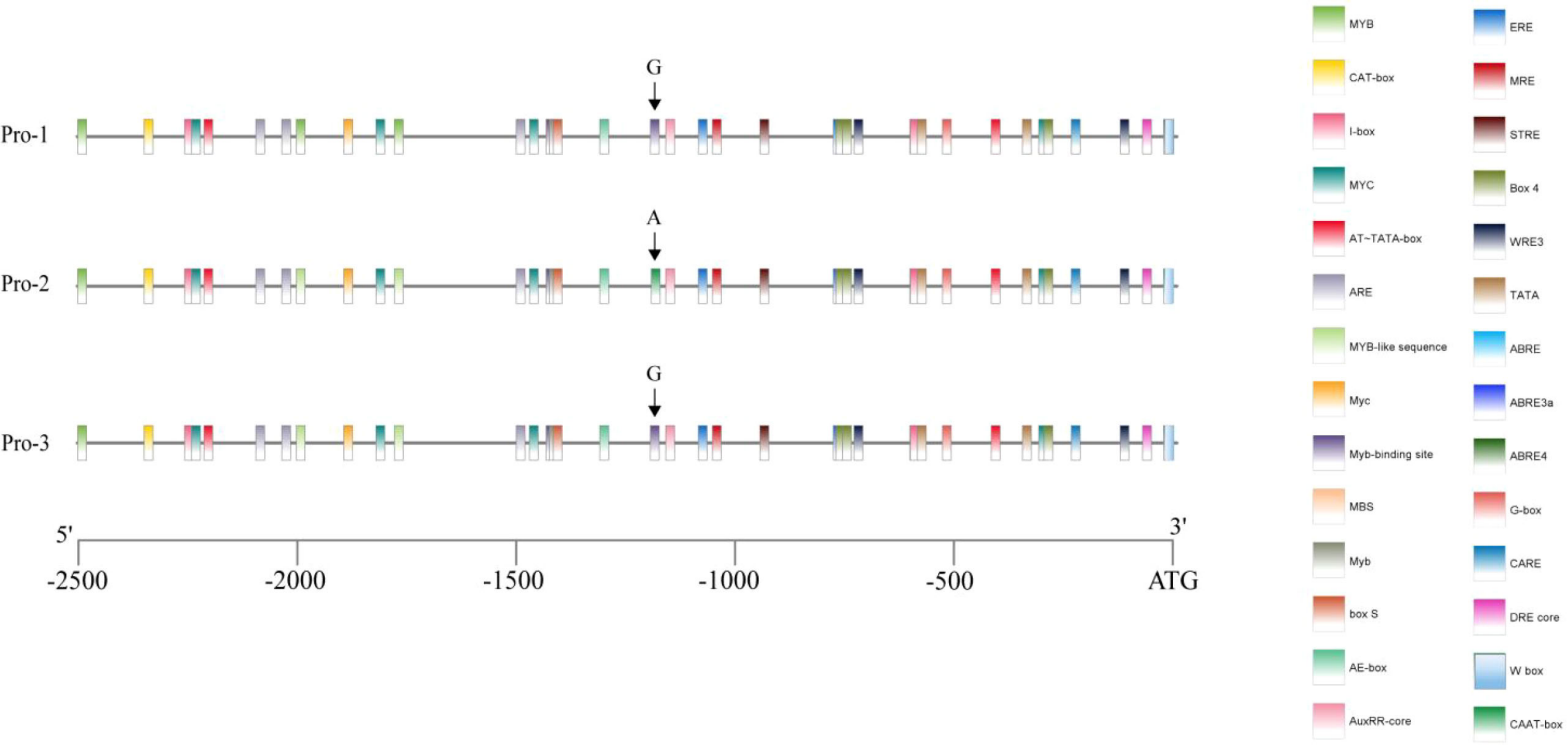
Promoter elements of candidate genes *Glyma.15G245300*.

The 2.5 kb upstream sequence from the start codon of the candidate gene *Glyma.15G245300* was analyzed to predict cis-regulatory elements in the promoter region across three genotypes using PlantCARE. The analysis revealed several gene-related elements, including auxin response elements (ARE), ethylene response elements (ERE), abscisic acid response elements (ABRE), and various transcription factor binding sites like MYC, MBS, and G-box. Moreover, stress response-related elements (STRE) were identified. A G-A mutation at position -1185 bp in the promoter region of the candidate gene led to the loss of a MYB cis-regulatory element and a Myb-binding site cis-regulatory element in Pro-2 type. However, this mutation introduced an additional CAAT-box cis-regulatory element (**Figure 6**).

Variations in the coding region of the *Glyma.15G245300* gene may result in alterations in the encoded protein, affecting both its tertiary structure and function. The amino acid sequence of the *Glyma.15G245300* gene was obtained from the Phytozome 13 database. In the RIL3613 population, a mutation was discovered at the 219th amino acid position, replacing alanine with serine, and at position 449, substituting aspartic acid with glutamic acid. Conversely, in the GP population, a mutation occurred at position 452, changing alanine to valine. Using Alphafold3, we predicted the tertiary structures of proteins Hap-1 and Hap-2 from the RIL3613 population, as well as CDS-1 and CDS-2 proteins from the GP population. The model with the highest confidence level was chosen for further analysis and visualized with PyMol software (**Supplementary Figure 4**). The results revealed that despite specific amino acid mutations, there were no significant differences in the tertiary structures of the *Glyma.15G245300* gene-encoded proteins, including Hap-1 and Hap-2 in the RIL3613 population, and CDS-1 and CDS-2 in the GP population.

## 4 Discussions

### 4.1 Advantages of using image recognition technology for disease resistance identification

The previous assessment of resistance to frogeye leaf spot in soybean varieties utilized disease indices for evaluating resistance levels, leading to subsequent classification based on these indices (Liu et al., 1991). However, the method based on inoculation faces significant variability due to differences in personnel operation, resulting in drawbacks such as extended assessment times, poor repeatability, and low accuracy. In this study, we employed the Labelme image recognition program to identify soybean leaf spots and calculate PDLA (Percentage Disease Leaf Area), enabling a more precise evaluation of disease resistance in soybean varieties. This approach allows for rapid identification of FLS (Frogeye Leaf Spot) on soybean leaves, offering enhanced accuracy compared to traditional methods and reducing human subjective bias in assessments. Particularly suitable for large-scale identification across multiple varieties, this technique effectively addresses various issues associated with conventional methods, enabling a more accurate assessment of variety resistance to FLS compared to traditional disease indices. Consequently, image recognition technology is increasingly integrated into the evaluation of FLS resistance (McDonald et al., 2023).

### 4.2 Detection power of FLS QTL in RIL3613

The size of the population is the key factor determining the detection efficacy of QTL, the accuracy of effect estimation, and the resolution ability of contribution rate. The larger the population, the higher the QTL detection efficacy, especially significantly increasing the detection rate of minor QTL; a small population is prone to miss minor loci, resulting in an underestimation of the number of QTL. For example, in the study of barley stripe rust, Vales et al. (2005) proposed that when the population size increased from 100 to 409, the number of detected QTL significantly increased. Major-effect QTL could be detected in a small population, while minor-effect QTL could only be stably detected in a large population. In the association study of soybean gray spot disease, a population of 202–250 materials could stably detect 11–12 resistance loci, with locus contribution rates ranging from 2.0% to 15.5% (Jiang et al., 2020; Di et al., 2021). In this study, the RIL3613 population contains 120 lines, which is indeed a relatively small size for QTL localization research in soybeans, potentially reducing the detection ability of minor-effect QTL. Therefore, the 18 QTN detected should be major-effect QTL.

Small populations often overestimate major-effect QTL (the “Beavis effect”), and the estimation of minor-effect QTL is unstable; large populations can more accurately restore the true effect and reduce false positives and biases (Concibido et al., 2004). For example, in the study of soybean cyst nematode QTL, Liu et al. (2015) proposed that in a 254 RIL population, major-effect QTL explained 29.5% of the variation, while minor-effect QTL accounted for only 5.5%–6.2%, and the effect estimation was more stable. In this study, due to the relatively small population size, the phenotypic variance explained rate (PVE) of some QTL may be overestimated, especially in the AC environment, where the PVE of qFLSm−18−1 is as high as 18.93%. This high contribution rate result requires vigilance against the positive bias brought by the Beavis effect. Subsequent verification in a larger population or in natural populations can be used to correct the effect values.

The larger the population, the more accurate the estimation of the phenotypic variance explained rate (PVE), and it can better distinguish major-effect and minor-effect loci. For example, in the study of soybean disease resistance QTL, a large population can stably distinguish contribution rate intervals of 5%–30%+, while a small population has difficulty accurately estimating minor-effect loci (<10%). Increasing the population size can increase the number of recombination events between markers and QTL, narrow the positioning interval, and improve resolution, laying the foundation for subsequent fine localization and gene cloning. In this study, the RIL3613 population consisted of 120 lines. For a QTL mapping study in soybeans, this is indeed a relatively small scale, which may reduce the detection ability of minor-effect QTL (PVE < 5%) and lead to an overestimation of the QTL effect. However, in this study, high-density interval mapping and multi-environment experimental designs were employed to determine phenotypes, which can ensure the reliability of high main-effect QTL detection and reduce false positives.

Numerous loci linked to resistance against frogeye leaf spot (FLS) have been documented in the literature (**Figure 2**). By McAllister et al. (2021) identified quantitative trait loci (QTLs) connected with FLS on chromosomes 13 and 19. McDonald et al. (2023) devised molecular markers for SNP loci situated on chromosome 11 to facilitate breeding efforts for enhancing resistance to frogeye leaf spot. Furthermore, Jiang et al. (2011) detected 5 simple sequence repeat (SSR) markers closely associated with *Rcs15*, a gene imparting resistance to frogeye leaf spot race 15.

Gu et al. (2021) conducted a genome-wide association study (GWAS) to pinpoint single nucleotide polymorphisms (SNPs) associated with FLS resistance on chromosomes 5 and 20. Sun et al. (2022) utilized GWAS and SNP methodologies to explore soybean’s physiological resistance against frogeye leaf spot race 1, unveiling 13 novel sites relevant to this resistance. Na et al. (2023) detected 15 quantitative trait nucleotides (QTNs) linked to resistance against FLS race 7. Dong et al. (2011) integrated the FLS race 7 resistance loci into the soybean Cregen genetic linkage map, thereby advancing our comprehension of genetic determinants influencing disease resilience in soybeans.

A comparison of the 19 QTL intervals identified in this study with the FLS resistance loci reported by previous researchers revealed that the qFEm-18–1 interval is situated near the AX-14428467 locus (Sun et al., 2022). Additionally, the qFLSm-15-2, qFLSm-15-3, and qFLSm-15–4 intervals are located in closer proximity to the Satt384 locus (Dong et al., 2011). The remaining QTLs were newly identified in this investigation.

### 4.3 Prediction of candidate genes for resistance to frogeye leaf spot

Previous studies have linked *Glyma.16G176800*, *Glyma.16G177300*, *Glyma.16G177400*, and *Glyma.16G182300* to resistance against FLS race 7 (Na et al., 2023). Furthermore, Sun et al. (2022) have shown the significance of *Glyma.05G121100*, *Glyma.17G228300*, *Glyma.19G006900*, and *Glyma.19G008700* in imparting resistance to FLS race 1 in soybeans. However, there is limited information on genes associated with resistance to mixed species of FLS. In this investigation, we identified 265 genes within 19 QTLs consistently mapped in two environments with narrow intervals using the recombinant inbred line RIL3613. Through functional annotation and comparative analysis of parental sequence variations, we identified *Glyma.15G245300* as a potential candidate linked to gray spot resistance. This gene is a member of the Cytochrome P450 family, a supergene family encoding monooxygenases present in animals, plants, microorganisms, and humans (Elfaki et al., 2018; Nebert et al., 1989). Cytochrome P450s (CYP450s) are crucial in various metabolic reactions in plants, primarily involved in the synthesis and metabolism of terpenoids, phenylpropanoids, alkaloids, sterols, fatty acids, plant hormones, and pigments. Cytochrome P450 enzymes play vital roles in regulating plant growth, development, and stress responses (Schuler and Werck-Reichhart, 2003). They are also involved in the modulation of plant hormones (Mizutani, 2012) and play a crucial part in the plant-pathogen interaction (Yan et al., 2016). Pathogens often induce indole-3-acetic acid (IAA) accumulation in host plants, which compromises the cell wall’s protective function (Li et al., 2015). Cytochrome P450 can counteract IAA accumulation through negative feedback regulation, thereby enhancing plant disease resistance. Dysregulated expression of CYP450 genes can disrupt the expression of cell wall-related genes, leading to changes in cell wall composition and impacting plant disease resistance (Wang et al., 2016). Moreover, upregulation of genes associated with the jasmonic acid/ethylene (JA/ET) signaling pathway can further enhance plant resistance (Yan et al., 2016).

Previous studies have mostly focused on the resistance of a single type of gray spot pathogen (*Cercospora sojina*) (Sun et al., 2022; Na et al., 2023). In contrast, this study used a mixture of the three main strains (1, 7, 15) from China, based on multi-environment stable QTL, sequence variations, haplotype analysis and expression patterns, and for the first time identified the cytochrome P450 gene as a key candidate gene for soybean resistance to gray spot disease. The reported resistance genes for gray spot disease are mostly NBS-LRR type disease proteins, kinases or transcription factors, which initiate immunity by recognizing pathogen effectors (Pham et al., 2015; Gu et al., 2021). However, *Glyma.15G245300* encodes a cytochrome P450 monooxygenase and participates in disease resistance through metabolic regulation, with a completely different mechanism of action. The expression profile of this gene shows significant upregulation at 3 h and 12 h after inoculation, indicating its involvement in the early basal immune response; while the known Rcs genes are mostly involved in late race-specific resistance. Furthermore, this study is the first to discover that the superior haplotype of *Glyma.15G245300* has been strongly selected during soybean domestication and breeding, suggesting that this gene mediates broad-spectrum, persistent, metabolic-type disease resistance, rather than merely race-specific allergic reactions. These results preliminarily reveal the potential role of this gene in enhancing plant disease resistance and provide a new target for broad-spectrum molecular breeding of soybean against gray spot disease.

## Conclusion

Nineteen quantitative trait loci (QTLs) were identified in the recombinant inbred line population RIL3613 using the Bin genetic map. Among them, eight QTLs were consistently detected across multiple environments and methodologies. Particularly, the gene *Glyma.15G245300* was implicated in conferring resistance to frogeye leaf spot. These results offer crucial insights for delving deeper into the genetic mechanisms of gray spot disease and are instrumental in advancing molecular breeding strategies to bolster resistance against frogeye leaf spot (FLS).

## Data Availability

All the data generated and analyzed in this study have been included in the article and its **Supplementary Materials**.
