# Supplementary material for: QTL mapping and key candidate gene identification of resistance to frogeye leaf spot (Cercospora sojina) in soybean
Source: Front Plant Sci. 2026 May 11;17:1773160. doi: 10.3389/fpls.2026.1773160 (PMC13199167; doi:10.3389/fpls.2026.1773160)
Supplement: Supplementary file 1 [file DataSheet1.pdf]

Supplementary Material

1 Supplementary Figures and Tables

1.1 Supplementary Figures

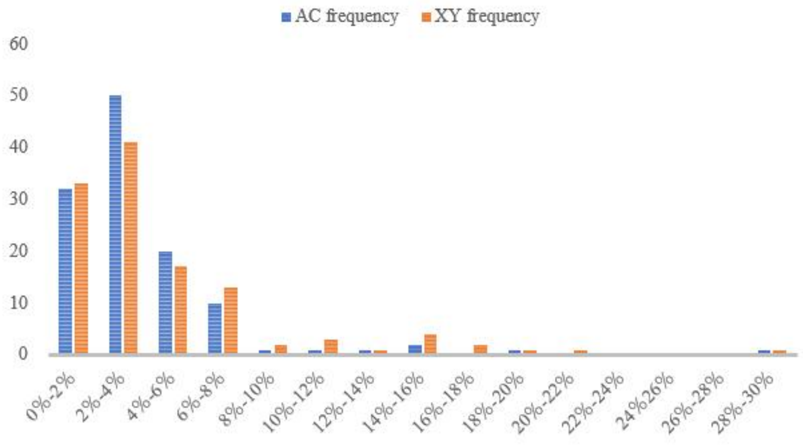

Supplementary FIGURE S1 Frequency distribution of relative lesion area of RIL3613 in two environment

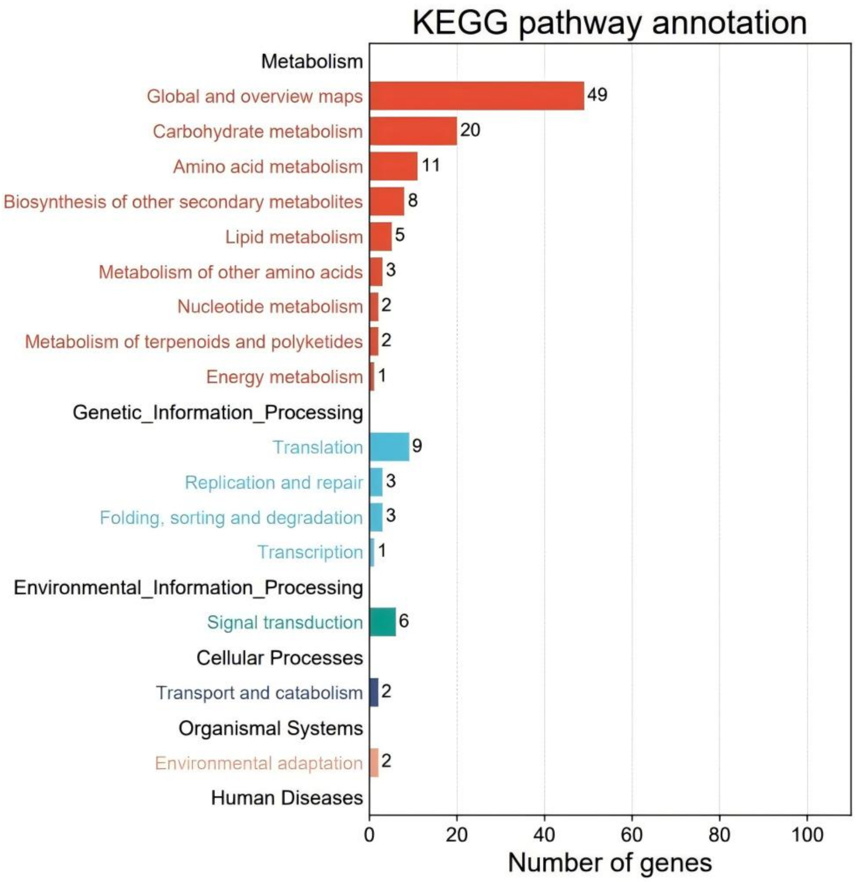

Supplementary FIGURE S2 KEGG annotation analysis of 265 genes of eight QTL

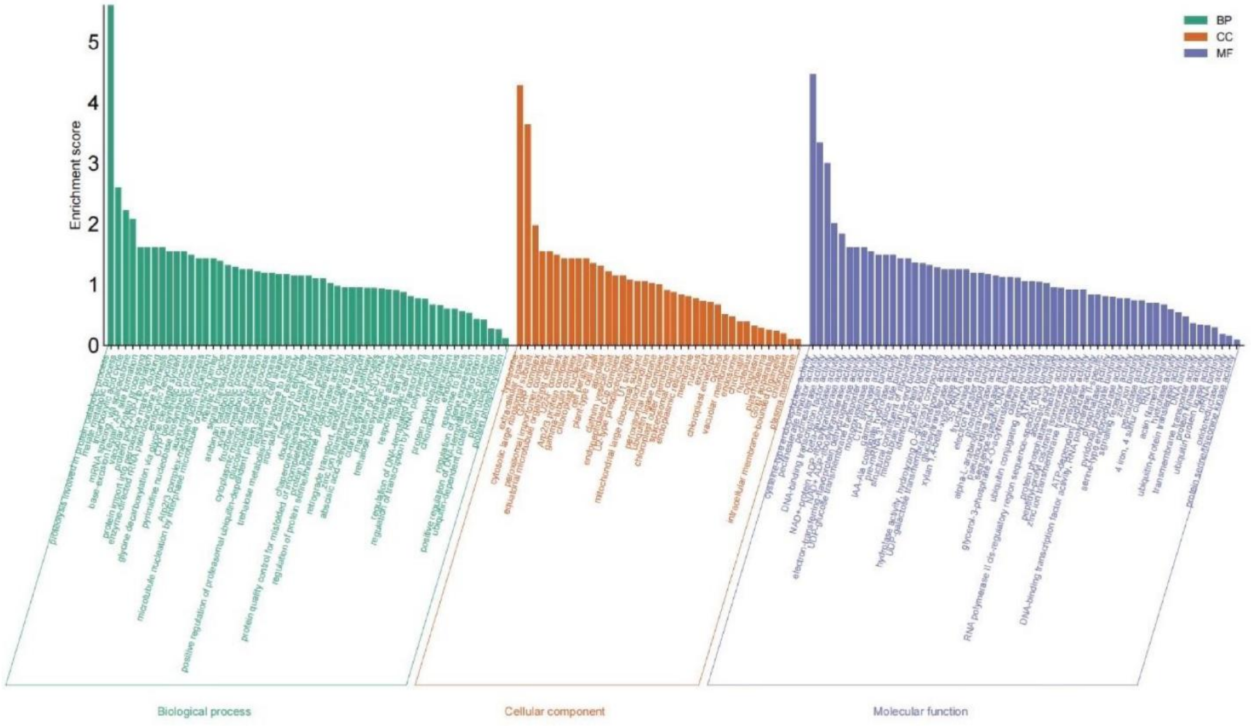

Supplementary FIGURE S3 GO enrichment analysis of 265 genes of eight QTL

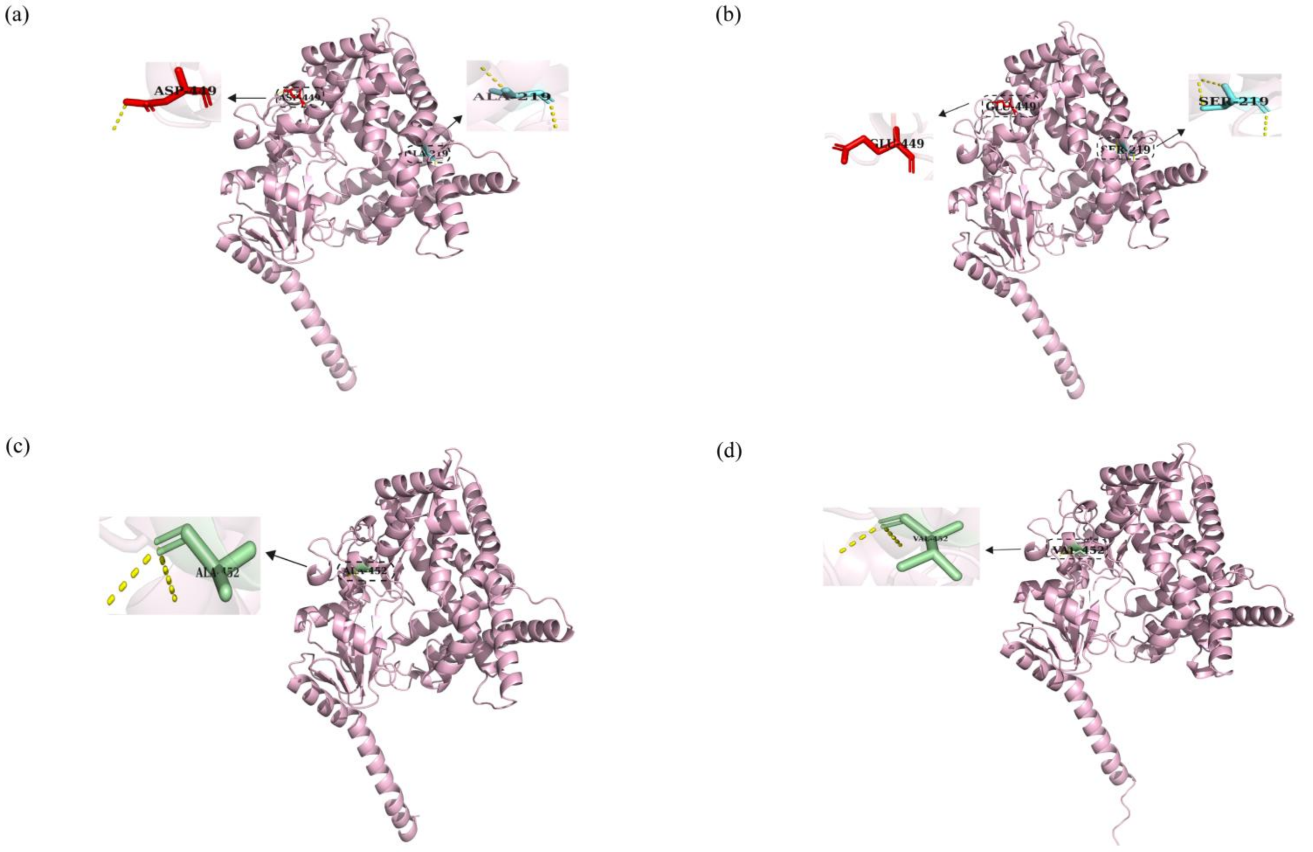

Supplementary FIGURE S4 The protein tertiary structure prediction of the different genotypes of *Glyma.15G245300* in the RIL3613 and the GP population. (a) The tertiary structure of the CDS-1 type protein in the RIL3613 population. (b) The tertiary structure of the CDS-2 type protein in the RIL3613 population. (c) The tertiary structure of the CDS-1 type protein in the GP population. (c) The tertiary structure of the CDS-2 type protein in the GP population.

1.2 Supplementary Tables

Supplementary TABLE S1 Primers used for cloning the gene *Glyma.15G24530*

Gene	Primer sequence
Forward Primer 5'-3'	TGGGAAGGGATGCTATCTTCAG
Reverse Primer 5'-3'	TGTCGGTAGCAACCTACAAAT

Supplementary TABLE S2 Information of 151 soybean resources in the Germplasm population (GP)

Cultivar	Source	Genotype		PDLA
		Promoter	CDS	
Heihe 42	Heihe Branch Institute of Heilongjiang Academy of Agricultural Sciences	GG	T	0.0258
Kenfeng 10	Institute of Crop Development, Heilongjiang Agricultural Reclamation Academy	GG	T	0.0152
Heihe 31	Heihe Branch Institute of Heilongjiang Academy of Agricultural Sciences	GG	T	0.0245
Heihe 25	Heihe Branch Institute of Heilongjiang Academy of Agricultural Sciences	GG	T	0.0361
Jiunong 29	Soybean Research Center of Jilin Academy of Agricultural Sciences	GG	T	0.0111
Dongnong 56	Northeast Agricultural University	/	T	0.0423
Jiyu 69	Soybean Research Center of Jilin Academy of Agricultural Sciences	/	T	0.0327
Hefeng 30	Hejiang Agricultural Science Research Institute of Heilongjiang Academy of Agricultural Sciences	GG	T	0.0034
Kenfeng 16-1	Institute of Crop Development, Heilongjiang Agricultural Reclamation Academy	GG	T	0.0185
Henong 60	Jiamusi Branch of Heilongjiang Academy of Agricultural Sciences	GG	T	0
Beidou 41	Beian Agricultural Science Research Institute of Heilongjiang Provincial Reclamation Bureau	GG	/	0.0199
Jiunong 21	Soybean Research Center of Jilin Academy of Agricultural Sciences	GG	T	0.0249
Fengshou 11	Keshan Agricultural Science Research Institute of Heilongjiang Academy of Agricultural Sciences	GG	/	0.015
Heihe 49	Heihe Branch Institute of Heilongjiang Academy of Agricultural Sciences	GG	T	0.0032

Beidou 10	Beian Agricultural Science Research Institute of Heilongjiang Provincial Reclamation Bureau	/	T	0.0258
Heihe 27	Heihe Branch Institute of Heilongjiang Academy of Agricultural Sciences	GG	T	0.0298
Kenfeng 11	Institute of Crop Development, Heilongjiang Agricultural Reclamation Academy	GG	T	0.0365
Kenfeng 6	Institute of Crop Development, Heilongjiang Agricultural Reclamation Academy	GG	T	0.0146
Hefeng 29	Hejiang Agricultural Science Research Institute of Heilongjiang Academy of Agricultural Sciences	GG	T	0.0055
Dongnong 49	Northeast Agricultural University	GG	T	0.0216
Dongnong 57	Northeast Agricultural University	GG	T	0.0213
Dongnong 47	Northeast Agricultural University	GG	/	0.0259
Kenfeng 14	Institute of Crop Development, Heilongjiang Agricultural Reclamation Academy	GG	/	0.0365
Nenfeng 4	Nenjiang Agricultural Research Institute of Heilongjiang Academy of Agricultural Sciences	GG	/	0.0677
Heinong 41	Institute of Soybean of Heilongjiang Academy of Agricultural Sciences	GG	T	0.0152
Dongnong 54	Northeast Agricultural University	GG	T	0.0555
Heihe 19	Heihe Branch Institute of Heilongjiang Academy of Agricultural Sciences	GG	T	0.0237
Heihe 12	Heihe Branch Institute of Heilongjiang Academy of Agricultural Sciences	GG	T	0.0478
Beifeng 11	Beian Agricultural Science Research Institute of Heilongjiang Provincial Reclamation Bureau	GG	T	0.0243
Heinong 11	Institute of Soybean of Heilongjiang Academy of Agricultural Sciences	/	T	0.0267
Hefeng 37	Hejiang Agricultural Science Research Institute of Heilongjiang Academy of Agricultural Sciences	GG	T	0.0166
Suinong 17	Suihua Branch, Heilongjiang Academy of Agricultural Sciences	GG	T	0.0358
Heinong 34	Institute of Soybean of Heilongjiang Academy of Agricultural Sciences	GG	/	0.0283
Beidou 34	Beian Agricultural Science Research Institute of Heilongjiang Provincial Reclamation Bureau	GG	/	0.0283
Nenao 5	Nenjiang County Yuandong Seed Industry Co., Ltd.	GG	T	0.0031
Zhonghuang 68	Institute of Crop Sciences of Chinese Academy of Agricultural Sciences	GG	T	0.0057

Kendou 26	Keshan Branch of Heilongjiang Academy of Agricultural Sciences	GG	T	0.0262
Heinong 44	Institute of Soybean of Heilongjiang Academy of Agricultural Sciences	GG	T	0.029
Beidou 14	Beian Agricultural Science Research Institute of Heilongjiang Provincial Reclamation Bureau	GG	T	0.0775
Heinong 55	Institute of Soybean of Heilongjiang Academy of Agricultural Sciences	/	T	0.0291
Heihe 44	Heihe Branch Institute of Heilongjiang Academy of Agricultural Sciences	GG	T	0.0009
Heihe 45	Heihe Branch Institute of Heilongjiang Academy of Agricultural Sciences	GG	T	0.0009
Beidou 49	Beian Agricultural Science Research Institute of Heilongjiang Provincial Reclamation Bureau	GG	T	0.0153
Heihe 28	Heihe Branch Institute of Heilongjiang Academy of Agricultural Sciences	GG	T	0.0389
Heihe 39	Heihe Branch Institute of Heilongjiang Academy of Agricultural Sciences	GG	T	0.0314
Mengdou 9	Hulunbuir Agricultural Science Research Institute	GG	T	0.0365
Beidou 40	Beian Agricultural Science Research Institute of Heilongjiang Provincial Reclamation Bureau	GG	T	0.0214
Nenfeng 15	Nenjiang Agricultural Research Institute of Heilongjiang Academy of Agricultural Sciences	GG	T	0.0565
Beidou 16	Beian Agricultural Science Research Institute of Heilongjiang Provincial Reclamation Bureau	GG	T	0.0224
Kendou 43	Institute of Crop Development, Heilongjiang Agricultural Reclamation Academy	GG	T	0.024
Hefeng 35	Hejiang Agricultural Science Research Institute of Heilongjiang Academy of Agricultural Sciences	GG	T	0.0721
Heinong 48	Institute of Soybean of Heilongjiang Academy of Agricultural Sciences	GG	T	0.0476
Heihe 35	Heihe Branch Institute of Heilongjiang Academy of Agricultural Sciences	GG	T	0.0359
Heihe 18	Heihe Branch Institute of Heilongjiang Academy of Agricultural Sciences	GG	T	0.0246
Heihe 14	Heihe Branch Institute of Heilongjiang Academy of Agricultural Sciences	GG	T	0.0207
Heinong 38	Institute of Soybean of Heilongjiang Academy of Agricultural Sciences	GG	T	0.0303
Keshan 1-2	Keshan Branch of Heilongjiang Academy of Agricultural Sciences	/	T	0.0428
Heihe 20	Heihe Branch Institute of Heilongjiang Academy of Agricultural Sciences	GG	T	0.0411

Fengshou 25	Keshan Agricultural Science Research Institute of Heilongjiang Academy of Agricultural Sciences	GG	T	0.0204
Hefeng 41	Hejiang Agricultural Science Research Institute of Heilongjiang Academy of Agricultural Sciences	GG	T	0.0065
Fengshou 24 -1	Keshan Agricultural Science Research Institute of Heilongjiang Academy of Agricultural Sciences	GG	T	0.0193
Dongnong 53	Northeast Agricultural University	/	T	0.0429
Jilin 35	Soybean Research Center of Jilin Academy of Agricultural Sciences	GG	T	0.0436
Heinong 64	Institute of Soybean of Heilongjiang Academy of Agricultural Sciences	GG	/	0.0188
Hefeng 25	Hejiang Agricultural Science Research Institute of Heilongjiang Academy of Agricultural Sciences	GG	T	0.0451
Heinong 52	Institute of Soybean of Heilongjiang Academy of Agricultural Sciences	GG	T	0.0342
Dongnong 42	Northeast Agricultural University	GG	T	0.0522
Kenfeng 13	Institute of Crop Development, Heilongjiang Agricultural Reclamation Academy	GG	T	0.0564
Kendou 32	Keshan Branch of Heilongjiang Academy of Agricultural Sciences	GG	T	0.0213
Mengdou 30	Hulunbuir Agricultural Science Research Institute	/	T	0.0432
Kendou 34	Keshan Branch of Heilongjiang Academy of Agricultural Sciences	GG	T	0.0135
Hefeng 55	Hejiang Agricultural Science Research Institute of Heilongjiang Academy of Agricultural Sciences	/	T	0.0365
Suinong 18	Suihua Branch, Heilongjiang Academy of Agricultural Sciences	GG	T	0.028
Hefeng 39	Hejiang Agricultural Science Research Institute of Heilongjiang Academy of Agricultural Sciences	GG	T	0.0031
Heihe 24	Heihe Branch Institute of Heilongjiang Academy of Agricultural Sciences	GG	T	0.0135
Heinong 35	Institute of Soybean of Heilongjiang Academy of Agricultural Sciences	GG	T	0.0455
Mengdou 11	Hulunbuir Agricultural Science Research Institute	GG	T	0.0635
Mufeng 1-2	Heilongjiang Academy of Agricultural Sciences,	GG	/	0.0465
Suinong 14-1	Suihua Branch, Heilongjiang Academy of Agricultural Sciences	GG	T	0.0482
Hefeng 38	Hejiang Agricultural Science Research Institute of Heilongjiang Academy of Agricultural Sciences	/	T	0.0313

Heihe1-1	Heihe Branch Institute of Heilongjiang Academy of Agricultural Sciences	GG	T	0.0954
Dongnong 52	Northeast Agricultural University	/	T	0.0297
Heihe 11	Heihe Branch Institute of Heilongjiang Academy of Agricultural Sciences	GG	T	0.0239
Fengshou 6	Keshan Agricultural Science Research Institute of Heilongjiang Academy of Agricultural Sciences	GG	T	0.0394
Mengdou 14	Hulunbuir Agricultural Science Research Institute	GG	T	0.0624
Kennong 4	Institute of Crop Development, Heilongjiang Agricultural Reclamation Academy	GG	T	0.0611
Heinong 61	Institute of Soybean of Heilongjiang Academy of Agricultural Sciences	GG	T	0.0627
Hongfeng 11	Hongxinglong Agricultural Administration Bureau of the General Administration of State-owned Farms of Heilongjiang Province, Agricultural Research Institute	GG	T	0.0548
Heihe 6	Heihe Branch Institute of Heilongjiang Academy of Agricultural Sciences	GG	T	0.0684
Heihe 53	Heihe Branch Institute of Heilongjiang Academy of Agricultural Sciences	GG	T	0.0622
Hefeng 45	Hejiang Agricultural Science Research Institute of Heilongjiang Academy of Agricultural Sciences	GG	T	0.0092
Heihe 5	Heihe Branch Institute of Heilongjiang Academy of Agricultural Sciences	GG	T	0.0182
Kendou 36	Keshan Branch of Heilongjiang Academy of Agricultural Sciences	GG	T	0.0276
Mengdou 12	Hulunbuir Agricultural Science Research Institute	GG	T	0.0458
Heinong 68	Institute of Soybean of Heilongjiang Academy of Agricultural Sciences	/	T	0.03
Suinong 23	Suihua Branch, Heilongjiang Academy of Agricultural Sciences	GG	T	0.0439
Hefeng 40	Hejiang Agricultural Science Research Institute of Heilongjiang Academy of Agricultural Sciences	GG	T	0.0278
Zihua 4-1	Landrace	/	T	0.0312
Heihe 46	Heihe Branch Institute of Heilongjiang Academy of Agricultural Sciences	/	T	0.019
Hefeng 36	Hejiang Agricultural Science Research Institute of Heilongjiang Academy of Agricultural Sciences	GG	T	0.0031
Shengdou 15	Heilongjiang Shengfeng Seed Industry Co., Ltd.	GG	T	0.0728
Dongnong 51	Northeast Agricultural University	GG	T	0.0918

Dongnong 50	Northeast Agricultural University	GG	T	0.1159
Dongsheng 1	Northeast Institute of Geography and Agroecology, Chinese Academy of Sciences	/	T	0.0656
Silijia	Landrace	GG	T	0.0262
Heihe 34	Heihe Branch Institute of Heilongjiang Academy of Agricultural Sciences	AG	A	0.0178
Heihe 40	Heihe Branch Institute of Heilongjiang Academy of Agricultural Sciences	AG	A	0.0389
Kenfeng 5	Mudanjiang Branch of Institute of Heilongjiang Agricultural Reclamation Academy	AG	A	0.0132
Mengdou 36-1	Hulunbuir Agricultural Science Research Institute	AG	A	0.0111
Heihe 33	Heihe Branch Institute of Heilongjiang Academy of Agricultural Sciences	AG	A	0.0122
Heihe 10	Heihe Branch Institute of Heilongjiang Academy of Agricultural Sciences	AG	A	0.0207
Heihe 37	Heihe Branch Institute of Heilongjiang Academy of Agricultural Sciences	AG	A	0.0179
Heihe 21	Heihe Branch Institute of Heilongjiang Academy of Agricultural Sciences	AG	A	0.0298
Heihe 38	Heihe Branch Institute of Heilongjiang Academy of Agricultural Sciences	AG	A	0.0041
Kenfeng 20	Institute of Crop Development, Heilongjiang Agricultural Reclamation Academy	AG	A	0.0316
Heihe 7	Heihe Branch Institute of Heilongjiang Academy of Agricultural Sciences	AG	A	0.0198
Heihe 15	Heihe Branch Institute of Heilongjiang Academy of Agricultural Sciences	AG	/	0.0381
Heinong 37-1	Institute of Soybean of Heilongjiang Academy of Agricultural Sciences	AG	A	0.031
Kenfeng 17	Institute of Crop Development, Heilongjiang Agricultural Reclamation Academy	AG	A	0.0311
Kendou 39	Keshan Branch of Heilongjiang Academy of Agricultural Sciences	/	A	0.0049
Beidou 19	Beian Agricultural Science Research Institute of Heilongjiang Provincial Reclamation Bureau	AG	A	0.0532
Heihe 54	Heihe Branch Institute of Heilongjiang Academy of Agricultural Sciences	AG	A	0.0394
Heihe 22	Heihe Branch Institute of Heilongjiang Academy of Agricultural Sciences	AG	A	0.0194
Heihe 30	Heihe Branch Institute of Heilongjiang Academy of Agricultural Sciences	AG	A	0.0199
Heihe 47	Heihe Branch Institute of Heilongjiang Academy of Agricultural Sciences	AG	A	0.0299

Kendou 28	Keshan Branch of Heilongjiang Academy of Agricultural Sciences	AG	A	0.0239
Heihe 36	Heihe Branch Institute of Heilongjiang Academy of Agricultural Sciences	AG	A	0.0191
Hefeng 43	Hejiang Agricultural Science Research Institute of Heilongjiang Academy of Agricultural Sciences	AG	A	0.0033
Hefeng 47	Hejiang Agricultural Science Research Institute of Heilongjiang Academy of Agricultural Sciences	AG	A	0.0065
Kennong 31	Institute of Crop Development, Heilongjiang Agricultural Reclamation Academy	/	A	0.0565
Heihe 8	Heihe Branch Institute of Heilongjiang Academy of Agricultural Sciences	AG	A	0.0586
Henong 75	Jiamusi Branch of Heilongjiang Academy of Agricultural Sciences	AG	A	0.0398
Kenfeng 22	Institute of Crop Development, Heilongjiang Agricultural Reclamation Academy	AG	A	0.0193
Hefeng 50	Hejiang Agricultural Science Research Institute of Heilongjiang Academy of Agricultural Sciences	AG	A	0.0065
Heihe 17	Heihe Branch Institute of Heilongjiang Academy of Agricultural Sciences	AG	A	0.0254
Shishengchangye	Imported from Japan	GA	T	0.0265
Jiyu 88	Soybean Research Center of Jilin Academy of Agricultural Sciences	GA	/	0.0286
Nenfeng 16	Nenjiang Agricultural Research Institute of Heilongjiang Academy of Agricultural Sciences	GA	T	0.2342
Heinong 43	Institute of Soybean of Heilongjiang Academy of Agricultural Sciences	GA	T	0.0477
Kennong 28	Institute of Crop Development, Heilongjiang Agricultural Reclamation Academy	GA	T	0.0325
Kendou 30	Keshan Branch of Heilongjiang Academy of Agricultural Sciences	GA	T	0.0298
Dongnong 48	Northeast Agricultural University	GA	T	0.0389
Heinong 40	Institute of Soybean of Heilongjiang Academy of Agricultural Sciences	GA	/	0.0225
Kenfeng 23	Institute of Crop Development, Heilongjiang Agricultural Reclamation Academy	GA	T	0.0516
Heinong 54	Institute of Soybean of Heilongjiang Academy of Agricultural Sciences	GA	T	0.0564
Heinong 56	Institute of Soybean of Heilongjiang Academy of Agricultural Sciences	GA	T	0.0709
Dongnong 55	Northeast Agricultural University	GA	T	0.0976

Suinong 4-1	Suihua Branch, Heilongjiang Academy of Agricultural Sciences	GA	T	0.091
Suinong 33-1	Suihua Branch, Heilongjiang Academy of Agricultural Sciences	GA	T	0.0053
Nenfeng 18	Nenjiang Agricultural Research Institute of Heilongjiang Academy of Agricultural Sciences	GA	T	0.0811
Suinong 30-1	Suihua Branch, Heilongjiang Academy of Agricultural Sciences	GA	T	0.0113

Supplementary TABLE S3 Primer sequence of RT-qPCR

Gene	Forward Primer 5’-3’	Reverse Primer 5’-3’
Glyma.15G245300	GAAAGCAATGAAGGCCGGT	TTGGCTAGTCATTGCAACGGT
TUA5	TGCCACCATCAAGACTAAGAGG	ACCACCAGGAACAACAGAAGG
